# Supplementary material for: search.bioPreprint: a discovery tool for cutting edge, preprint biomedical research articles
Source: F1000Res. 2016 Jul 20;5:1396. Originally published 2016 Jun 16. [Version 2] doi: 10.12688/f1000research.8798.2 (PMC4957174; doi:10.12688/f1000research.8798.2)
Supplement: Peer review history

# Peer review: Referee response for version 2

**DOI:** 10.5256/f1000research.9946.r15095

**Published:** 2016-07-21

**Authors:** Prachee Avasthi

**Affiliations:** 1 Department of Anatomy and Cell Biology, University of Kansas Medical Center, Kansas City, KS, USA

The authors have adequately addressed my comments. It is interesting to note that a Google Scholar search for "[search term] preprint" does not restrict results exclusively to preprints. While an advanced search in Google Scholar for articles published in only preprint servers would likely circumvent this, it is far more cumbersome to list all preprint servers in a Google Scholar advanced search (along with any new preprint servers that are developed) than to use search.Biopreprint (which will do this automatically). With the addition of a sort by date feature, this tool and associated bookmarklet will be an essential addition to the workflow of researchers.

I have read this submission. I believe that I have an appropriate level of expertise to confirm that it is of an acceptable scientific standard.

# Peer review: Referee response for version 1

**DOI:** 10.5256/f1000research.9471.r14408

**Published:** 2016-07-06

**Authors:** Cynthia Wolberger

**Affiliations:** 1 Department of Biophysics and Biophysical Chemistry, School of Medicine, Johns Hopkins University School of Medicine, Baltimore, MD, USA

This article contains a thorough description of a new search tool, search.bioPreprint, that can be used to search multiple preprint archives using keywords. In light of the existence of multiple preprint servers that can be used to post preprints in the biological sciences, the development of a tool that enables full-text searching of all current preprint archives (arXiv, bioRxiv, F1000Research, PeerJ Preprints) is a welcome one. The search capabilities of search.bioPreprint and the bookmarklet app are well-described.

 Looking ahead, the authors will hopefully consider further improvements to the search site. Better documentation on the search site itself explaining how results are returned and ranked would be helpful. This reviewer quickly learned that the search returns approximate word match results, not just exact matches; this should be clarified on the web site. Moreover, approximate matches sometimes appear be ranked more highly than exact matches; the reason for this should be examined and remedied, if possible. It would also be helpful to have options to rank results in other ways, in particular, by date.

I have read this submission. I believe that I have an appropriate level of expertise to confirm that it is of an acceptable scientific standard.

# Response

**Published:** 2016-07-13

**Authors:** Ansuman Chattopadhyay

**Affiliations:** Health Science Library System, University of Pittsburgh, USA

We sincerely thank the reviewer for considering the manuscript as “Approved” and for the constructive review.

**Reviewer Comment: **“…the authors will hopefully consider further improvements to the search site. Better documentation on the search site itself explaining how results are returned and ranked would be helpful. “

**Author’s Response:**

 We appreciate the  suggestions and will take measures to improve documentation on the search site.

**Reviewer Comment:** “This reviewer quickly learned that the search returns approximate word match results, not just exact matches; this should be clarified on the web site. Moreover, approximate matches sometimes appear be ranked more highly than exact matches; the reason for this should be examined and remedied, if possible.”

**Author’s Response:**

 We agree that better documentation is needed and will add a thorough description of the search process on the search site. One of the limitations of this search engine is it completely depends on the search prowess of the individual sources and we do not have any control over that. If the query term is placed within quotation marks, it forces the search engines to apply an exact word matching algorithm thus mitigating the issue of approximate word matching.

**Reviewer Comment:** “It would also be helpful to have options to rank results in other ways, in particular, by date.”

**Author’s Response:**

 We acknowledge the pressing need for an option to sort preprint articles by date as both reviewers mentioned it. We are actively working to bring this functionality into search.bioPreprint.

# Peer review: Referee response for version 1

**DOI:** 10.5256/f1000research.9471.r14404

**Published:** 2016-07-04

**Authors:** Prachee Avasthi

**Affiliations:** 1 Department of Anatomy and Cell Biology, University of Kansas Medical Center, Kansas City, KS, USA

search.bioPreprint is a useful tool for the scientific community and this article nicely outlines the key features and use cases for the service. A particular strength of the tool is the site maintenance and quality control by the University of Pittsburgh Health Sciences Library System. Also, the flexibility to incorporate new preprint servers due to continued site support is a benefit. The development of a bookmarklet for this purpose is novel, convenient and very useful.

 I have a few comments for authors to address:

  

1. There is no mention in the abstract that preprints can be found through Google Scholar. While there is a temporary bug associated with Google Scholar’s treatment of subsequently published preprints (documented in the following blog posts by Dr. Wilke), preprints are indeed searchable across platforms through Google Scholar. Please mention this and note any benefits of your tool. Google Scholar is mentioned on p2 but only in the context of indexing peer reviewed publications.
  http://serialmentor.com/blog/2014/11/1/the-google-scholar-preprint-bug
  http://serialmentor.com/blog/2015/10/8/Google-Scholar-bug-redux
2. The search results on search.bioPreprint are ordered by relevance with no option to re-sort. After some time searching using search.bioPreprint, the need to sort results by date became quickly evident. Likely, many users will be interested to know what new preprints are available across platforms since a previous search. A search term on Google Scholar followed by “preprint” and one click on the “sort by date” link produces date-sorted preprints from multiple sources (biorxiv, arrive, peerj etc). Similarly, while prepubmed.org also does not have sort functionality, the default ordering appears to be by date. I am unsure how difficult sort functionality would be to implement but some mention of this issue and any plans to implement such a feature in the future is recommended.
3. Outside of benefits to readers, an additional benefit I can see to a cross-platform preprint search engine is that it becomes easier for journal editors to identify/solicit submission of preprints and for grant reviewers to find preprints prior to journal publication. While these are also benefits of preprints in general, which is outside the scope of this article, authors may want to include this additional motivation/rationale as these are also particular benefits of a cross-platform tool. For example, a reviewer may be more likely to read a pending publication if they don’t need to search many different sites.
4. It seems the mention of prepubmed.org in the text is limited to a discussion of priority. Since search.bioPreprint is sold as a one-stop shop, I would have instead liked to see a concise comparison with the other cross-platform searches (prepubmed and also Google Scholar) to help users clearly identify any feature differences or benefits of using search.Biopreprint. The unique features of search.Biopreprint are described throughout the article but a concise comparison or table of features/search result data would be advantageous to readers.
5. This article was posted on both F1000Research and bioRxiv. Interestingly, only the bioRxiv version appears as a search result on both search.bioPreprint and Google Scholar, while prepubmed finds both versions. I am curious if some modification by the quality check team would fix this problem or if there is some inherent limitation of search.bioPreprint for preprints posted on more than one server.

 

 Overall, this article clearly describes usage and features of a new tool for cross-platform preprint search that appears to have the advantages of continuous maintenance, useful topic filtering and associated bookmarklet.

I have read this submission. I believe that I have an appropriate level of expertise to confirm that it is of an acceptable scientific standard, however I have significant reservations, as outlined above.

# Response

**Published:** 2016-07-13

**Authors:** Ansuman Chattopadhyay

**Affiliations:** Health Science Library System, University of Pittsburgh, USA

We sincerely thank the reviewer for the constructive review and thoughtful suggestions. Specifically, we appreciate the comments that search.bioPreprint is “a useful tool for the scientific community” and that “the development of a bookmarklet is novel, convenient and very useful.” We submitted a revised version of our paper that addresses the reviewer’s concerns, and we hope the reviewer finds this improved version acceptable without further revision.

**Reviewer Comment 1:**

 There is no mention in the abstract that preprints can be found through Google Scholar. While there is a temporary bug associated with Google Scholar’s treatment of subsequently published preprints (documented in the following blog posts by Dr. Wilke), preprints are indeed searchable across platforms through Google Scholar. Please mention this and note any benefits of your tool. Google Scholar is mentioned on p2 but only in the context of indexing peer reviewed publications.

**Author Response:**

 Thanks for showing us the workaround  for retrieving preprint articles via Google Scholar (GS) by adding “preprint” as a text with the search term(s). We missed the preprint search capability of GS as it is not listed as a valid filter such as “include patents.”

 However, we noticed that the articles retrieved by a preprint search using GS are not always valid preprints. For example, a search for “ asthma preprint” retrieves many articles already published that were never available as a preprint. When the search results (2,430) are sorted by relevence, the third and fourth citations from the top, “Heliox vs air-oxygen mixtures for the treatment of patients with acute asthma: a systematic Overview” by AMH Ho etal. and “Genomic approaches to understanding Asthma” by LJ Palmer etal., are published articles from Elsevier journal Chest (vol 123, issue 3) and from the journal Genome Research (CSH Press), respectively. After investigating a few non-preprint articles, we found the reason for the inclusion of non-preprint published articles in the GS search result is either a mention of a preprint article in the reference list or in the acknowledgement section.

 The results for another search,“CRISPR preprint,” results in 472 articles by GS and here also the third citation from the top is a published paper from the Journal Genome Research (CSH Press).

 We added a few sentences on using GS to search for preprints under Conclusions:  “ Google Scholar (GS), a popular scholarly literature search engine that provides cross-discipline search functionality, does not include preprint articles as a filter option. Hence, many avid GS users try a workaround by including preprint with the query term, (E.g., “asthma preprint” or “CRISPR preprint”) with the assumption of retrieving only preprint articles fetched from major preprint servers. In contrast, the GS search results in a mixed population of articles comprising both actual preprints and peer-reviewed published articles in which the term “preprint” appears somewhere in the full text of the article.”

**Reviewer Comment 2:**

 The search results on search.bioPreprint are ordered by relevance with no option to re-sort. After some time searching using search.bioPreprint, the need to sort results by date became quickly evident. Likely, many users will be interested to know what new preprints are available across platforms since a previous search. A search term on Google Scholar followed by “preprint” and one click on the “sort by date” link produces date-sorted preprints from multiple sources (biorxiv, arrive, peerj etc). Similarly, while prepubmed.org also does not have sort functionality, the default ordering appears to be by date. I am unsure how difficult sort functionality would be to implement but some mention of this issue and any plans to implement such a feature in the future is recommended.

**Author Response:**

 We acknowledge the pressing need for an option to sort preprint articles by date as both reviewers mentioned it. We are actively working to bring this functionality into search.bioPreprint.

 We want to stress that the “Sort by date” feature offered by Google Scholar (GS) is abysmal. It drastically drops the number of retrieved articles compared to the default search results. For example, a GS search for“Asthma preprint” retrieves 2,430 citations by the default “Sort by relevence” option, but displays only 6 articles after selecting “Sort by date.” The same thing happens for another search: “Crispr preprint” – Sort by relevance=471; Sort by date=5.

 In the revised manuscript we mention the need for reordering the search results by date by adding a new paragraph at the end of the Limitations section: ”Currently, the search.bioPreprint default search results are ordered by relevance without any option to re-sort by date. The authors are aware of the pressing need for this added feature and if possible will incorporate it into the next version of the search tool.”

**Reviewer Comment 3:**

 Outside of benefits to readers, an additional benefit I can see to a cross-platform preprint search engine is that it becomes easier for journal editors to identify/solicit submission of preprints and for grant reviewers to find preprints prior to journal publication. While these are also benefits of preprints in general, which is outside the scope of this article, authors may want to include this additional motivation/rationale as these are also particular benefits of a cross-platform tool. For example, a reviewer may be more likely to read a pending publication if they don’t need to search many different sites.

**Author Response:**

 Thanks for the suggestion. We added a sentence under Conclusion: “Referees during the grant or journal article review process might also find this bookmarklet useful as it quickly retrieves pre-published articles via the cross-platform preprint search.”

   

**Reviewer Comment 4:** It seems the mention of prepubmed.org in the text is limited to a discussion of priority. Since search.bioPreprint is sold as a one-stop shop, I would have instead liked to see a concise comparison with the other cross-platform searches (prepubmed and also Google Scholar) to help users clearly identify any feature differences or benefits of using search.Biopreprint. The unique features of search.Biopreprint are described throughout the article but a concise comparison or table of features/search result data would be advantageous to readers.

**Author Response:**

 Thanks for the suggestion. Google Scholar does not provide an option to limit searches to preprint articles, and the workaround of including “preprint” with the query term results in a mixed population of articles comprising both actual preprints and peer-reviewed published articles. We are hesitant to consider Google Scholar as a preprint search engine and comparable to search.bioPreprint and PrePubMed.  The purpose of this article is to present search.bioPreprint as a means to locate preprint articles, and its release pre-dates PrePubMed.  We leave it to others to determine the pros and cons of using search.bioPreprint, and hope that they leave comments so we can improve the tool when possible.

**Reviewer Comment 5:**

 This article was posted on both F1000Research and bioRxiv. Interestingly, only the bioRxiv version appears as a search result on both search.bioPreprint and Google Scholar, while prepubmed finds both versions. I am curious if some modification by the quality check team would fix this problem or if there is some inherent limitation of search.bioPreprint for preprints posted on more than one server.

**Author Response:**

 We appreciate your efforts in discovering this search error. The Health Sciences Library System’s quality check team has investigated this issue and is working on a solution. We anticipate a quick fix of this problem.

# Response

**Published:** 2016-07-13

**Authors:** Ansuman Chattopadhyay

**Affiliations:** Health Science Library System, University of Pittsburgh, USA

Jordan ,

 We appreciate your careful reading of our article and clarification of PeerJ’s search limitations.

 In the latest version we revised the text to read “We want to acknowledge this new resource, but emphasize that search.bioPreprint offers not only full text searching (with the exception of  PeerJ Preprints), but also topical and source-based clustering of results. In addition, our tool has been available since mid-February 2016, around the same time as the ASAPbio meeting, where it was mentioned during discussions.”
